# Molecular identification and optimization of indole acetic acid production by *Fusarium oxysporum* AUMC 16,438 for biofertilizer application

**DOI:** 10.1038/s41598-026-35223-z

**Published:** 2026-01-27

**Authors:** Sodaf A. Maan, Sayeda A. Abdelhamid

**Affiliations:** 1 Department of Agricultural Microbiology, Faculty of Agriculture, Ain Shams University, Cairo, Egypt; 2 Microbial biotechnology Department, National Research Center, Giza, Egypt

**Keywords:** Indole acetic acid (IAA), Fusarium oxysporum, Rhizosphere fungi, Phytohormone production, OFAT optimization, Agro-industrial wastes, Biofertilizer, Biological techniques, Biotechnology, Microbiology, Plant sciences

## Abstract

Indole-3-acetic acid (IAA) is a key phytohormone that regulates essential plant physiological processes and enhances overall growth. This study aimed to produce and optimize IAA synthesis by a fungal isolate obtained from rhizosphere soils in Egypt. Twenty fungal isolates were screened for IAA production, and isolate FSA12 showed the highest yield (23.98 ± 1.98 µg/mL). Molecular identification based on ITS sequencing, supported by morphological characterization, revealed that FSA12 is most likely *Fusarium oxysporum* AUMC 16,438, and its sequence was deposited in GenBank (accession no. PP990199). Optimization using the OFAT approach indicated that the optimal L-tryptophan concentration, incubation temperature, pH, incubation period, and inoculum size were 0.6%, 30 °C, pH 6, 12 days, and three mycelial discs, respectively. To reduce production cost, several agro-industrial wastes were evaluated as alternative carbon sources; banana peel proved the most effective, likely due to its natural L-tryptophan content. Application of the produced IAA significantly enhanced wheat seed germination compared with the untreated control, demonstrating its potential use as an eco-friendly biofertilizer in sustainable agriculture.

**Supplementary Information:**

The online version contains supplementary material available at 10.1038/s41598-026-35223-z.

## Introduction

 Auxins are fundamental plant hormones that regulate key developmental processes, including cell elongation, cell division, root initiation, fruit development, and responses to environmental cues such as light and gravity^1–3^. Indole-3-acetic acid (IAA) is the most common and biologically active auxin, shaping plant morphology and coordinating critical processes in growth and development^4,5^. As a central molecular signal, IAA mediates plant responses to environmental stimuli, including phototropism, gravitropism, thigmotropism, and defense mechanisms^6,7^. Although synthetic auxin analogues like 1-naphthaleneacetic acid (NAA) are widely used in agriculture, their high cost, chemical instability, and potential health and environmental risks limit their safe and sustainable application^8,9^. This highlights a pressing need for eco-friendly alternatives.Microbial biosynthesis of IAA presents a promising, low-cost, and sustainable approach compared to chemical synthesis^10^. Notably, many microorganisms can produce IAA at rates exceeding those of plants, making them attractive candidates for biofertilizer development^11^. This functional trait is widespread across diverse taxa, including fungi (e.g., *Aspergillus*, *Fusarium*, and *Trichoderma*)^12,13^, yeasts^14^, and numerous plant-growth-promoting bacteria^15,16^. These microbes typically synthesize IAA via multiple pathways, most commonly through tryptophan-dependent mechanisms^17^. In the rhizosphere, a mutualistic relationship is established: plants exude organic compounds, such as tryptophan, which stimulate resident microbes to produce IAA, thereby enhancing root development, nutrient uptake, and overall plant productivity^1,18–20^. Microbial IAA production is strongly influenced by nutritional and environmental factors, including L-tryptophan availability, pH, temperature, and carbon sources^21^. With growing interest in circular bioeconomy principles, agro-industrial residues have emerged as valuable, cost-effective substrates for optimizing the fermentation and production of microbial metabolites like IAA^22^. Among IAA-producing fungi, *Fusarium oxysporum* is a ubiquitous soil- and rhizosphere-inhabiting species of particular interest due to its ecological duality, encompassing both pathogenic strains and non-pathogenic isolates that play vital roles in nutrient cycling and plant-microbe interactions^23^. Non-pathogenic *F. oxysporum* can colonize plant roots endophytically or in the rhizosphere, where they promote plant growth through various mechanisms, including the synthesis of phytohormones like IAA, improved nutrient solubilization, and induced stress tolerance^24^. Several studies confirm that various *Fusarium* species and *F. oxysporum* isolates can produce IAA and stimulate root elongation, lateral root formation, and seedling vigor in diverse crops^25,26^. However, these beneficial traits are highly strain-specific, necessitating careful characterization to distinguish growth-promoting isolates from pathogenic ones. *Fusarium oxysporum* AUMC 16,438 is a rhizosphere-derived isolate preserved in the Assiut University Mycological Centre (AUMC). Its potential for IAA production and plant growth promotion has not been investigated in detail. Therefore, the present study aimed to optimize indole-3-acetic acid production by *F. oxysporum* AUMC 16,438 and evaluate its biofertilizer potential using a wheat seed germination model. To the best of our knowledge, this is the first report detailing the IAA-producing capability of this specific strain, its systematic physiological optimization, and a direct assessment of its biological efficacy on crop seeds. By integrating strain characterization, process optimization, and functional bioassays, this work provides new insights into the agricultural application of IAA produced by *F. oxysporum* AUMC 16,438.

## Materials and methods

### Isolation of IAA-producing fungi

Four soil samples were collected from cultivated fields in Qalyubia Governorate, Egypt, and transported to the laboratory in sterile plastic bags under cooling conditions. Samples were stored at 6 °C until processing. Fungi were isolated using Potato Sucrose Agar (PSA), and plates were incubated for 7 days at 25 ± 2 °C. Emerging colonies were purified by repeated subculturing onto fresh PSA plates. Pure isolates were maintained on PSA slants at 4 ± 1 °C for subsequent analyses^27^.

### Screening for IAA production

Screening for IAA production was performed using Czapek’s Dox liquid medium supplemented with 0.2 g/L L-tryptophan. The medium contained (g/L): glucose (30), NaNO₃ (3), MgSO₄·7 H₂O (0.5), KH₂PO₄ (1.0), yeast extract (5.0), FeSO₄·7 H₂O (0.01), KCl (0.5), and L-tryptophan (0.2). The pH was adjusted to 5.0 prior to autoclaving at 121 °C for 20 min. Two 6-mm discs of each isolate were inoculated into 50 mL of production medium and incubated at 25 ± 2 °C on a rotary shaker (150 rpm) for 8 days. All experiments were conducted in triplicate^28^.

### Extraction of IAA

At the end of incubation, cultures were filtered through Whatman No. 113 filter paper, followed by membrane filtration through a 0.22 μm filter to obtain cell-free supernatants. Fungal biomass was washed, dried at 60 °C, and weighed to determine dry weight. IAA was extracted from supernatants using two volumes of ethyl acetate. Organic layers were evaporated using a rotary evaporator, and the residue was stored for subsequent analyses^29^.

### Quantification of IAA

IAA concentrations were first quantified using the Salkowski colorimetric assay^30^. One milliliter of supernatant was mixed with 2 mL of Salkowski reagent and incubated in the dark for 30 min at room temperature. Absorbance was measured at 530 nm and compared with a standard IAA calibration curve.Confirmation of IAA identity was performed by High Performance Liquid Chromatography (HPLC) using an Agilent ChemStation system equipped with an RP-C18 column (250 mm × 4 mm, 5 μm). The mobile phase consisted of acetic acid: water: acetonitrile (1:15:85, v/v), with a flow rate of 1.0 mL/min. Detection was conducted at 254 nm, and peak areas were compared with authentic IAA standards (Sigma, USA)^31^.

### Qualitative detection of Mycotoxins using Thin-Layer chromatography (TLC)

Culture extracts were screened for mycotoxin production using thin-layer chromatography following the method of Chatterjee et al.^32^. Analyses were performed at the Animal Health Research Institute, Agricultural Research Center, Egypt.

### Morphological and molecular identification of the IAA-producing isolate

Morphological identification was performed using slide culture preparations following the method of Booth^33^, Slides were examined under a Zeiss Axiostar Plus Trinocular Microscope at 1000× magnification and photographed using a PowerShot G6 digital camera (Canon, Japan). For scanning electron microscopy (SEM), fungal mycelia were carefully harvested and fixed in 2.5% glutaraldehyde prepared in 0.1 M phosphate buffer (pH 7.2) for 12 hours at 4°C. The samples were then washed three times with the same buffer and dehydrated through an ethanol for 10–15 minutes. The dehydrated samples were air-dried, mounted onto aluminum stubs using double-sided conductive carbon tape, and sputter-coated with a thin layer of gold to enhance conductivity^34^. The prepared specimens were subsequently examined using a JEOL JSM-5300LV scanning electron microscope at 7500× magnification. Identification was further supported by comparison with the *Pictorial Atlas of Soil and Seed Fungi*.”

For molecular identification, genomic DNA was extracted from 4-day-old cultures grown in Czapek’s Dox medium according to Sharma et al.^35^. The extracted genomic DNA met the quality requirements for downstream PCR amplification, showing an A260/280 ratio within the acceptable purity range (1.8–2.0) and a measurable concentration sufficient for ITS amplification, as determined using a NanoDrop spectrophotometer. The ITS region was amplified using primers ITS1 (5′-TCCGTAGGTGAACCTGCGG-3′) and ITS4 (5′-TCCTCCGCTTATTGATATGC-3′). PCR conditions included initial denaturation at 95 °C for 10 min; 35 cycles of 95 °C for 30 s, 57 °C for 60 s, and 72 °C for 90 s; followed by a final extension at 72 °C for 10 min. Purified amplicons were sequenced using the PRISM BigDye Terminator v3.1 Kit and analyzed on an ABI Prism 3730XL DNA Analyzer. Sequences were compared with NCBI BLAST, and phylogenetic relationships were inferred. The sequence was deposited in GenBank and assigned an accession number.

### Optimization of cultural conditions for IAA production

Optimization was carried out using one-factor-at-a-time (OFAT) experiments. Cultures were incubated in 250 mL Erlenmeyer flasks containing 50 mL of Czapek’s Dox medium supplemented with L-tryptophan and maintained at 150 rpm under continuous light. After incubation, IAA concentration, fungal biomass, and productivity were determined for all treatments.

### Effect of L-tryptophan concentration

L-tryptophan levels of 0.1–1.5% were tested. Media were adjusted to pH 5.0 and incubated at 25 ± 2 °C for 8 days.

#### Effect of incubation temperature

Cultures were incubated at 20, 25, 30, 35, and 40 °C for 8 days.

#### Effect of initial pH

The medium pH was adjusted to 4, 5, 6, 7, or 8 prior to sterilization, and cultures were incubated for 8 days.

#### Effect of incubation period

Incubation periods ranging from 6 to 18 days were evaluated to determine the optimum harvest time.

#### Effect of inoculum size

Different inoculum sizes (1–5 mycelial discs, 6 mm each) were tested to determine the optimal biomass for maximum IAA synthesis.

### Screening of agro-industrial wastes for IAA production

To identify a low-cost substrate for scalable IAA production, six agro-industrial residues—sweet whey, banana peels, orange peels, wheat straw, wheat bran, and sugarcane bagasse—were screened. Solid substrates were washed, air-dried, oven-dried at 60 °C to constant weight, milled, and sieved to obtain a homogeneous particle size of ~ 2 mm. Fermentation media were prepared by supplementing the defined basal salts medium (see Sect. 2.1) with 5% (w/v) of each substrate. A medium containing only basal salts served as the negative control. The concentration of 5% was chosen based on preliminary trials and literature data, providing sufficient nutrients for fungal growth without inhibitory effects. A standardized spore inoculum of *F. oxysporum* AUMC 16,438 was prepared as described in Sect. 2.2. Flasks containing 100 mL of production medium were inoculated with 5% (v/v) of the spore suspension (4.2 × 10⁶ spores/mL). Cultures were incubated at 30 °C with agitation at 150 rpm for 12 days. All conditions were tested in triplicate as independent biological replicates. After fermentation, cultures were vacuum-filtered. The mycelial biomass was washed, oven-dried at 70 °C, and weighed to determine dry cell weight (DCW). The cell-free filtrate was centrifuged at 10,000 × g for 10 min, and the supernatant was stored at − 20 °C until analysis. IAA concentration was determined as previously described. IAA yield (mg/L) and specific productivity (mg IAA per g DCW per day) were calculated to evaluate substrate performance.

### Application of fungal IAA in wheat seed germination

Wheat (*Triticum aestivum* L.) was selected as the test plant due to its global agronomic importance, well-documented sensitivity to auxins, and frequent use as a model cereal crop in seed germination and biofertilizer evaluation studies. The bioactivity of fungal-derived IAA was assessed using the standard blotter method^36^. Wheat seeds were surface-sterilized in 0.1% HgCl₂ for 5 min, rinsed thoroughly with sterile distilled water (3–5 washes), and air-dried under aseptic conditions. Seeds were then soaked for 6 h in fungal IAA solutions at three concentrations (25, 50, and 100 µg/mL), which were selected based on preliminary trials and literature reports to cover a range of biologically relevant doses. To specifically attribute observed growth effects to IAA, the study included two types of controls: (i) seeds soaked in sterile distilled water (negative control) to account for baseline growth without exogenous auxin, and (ii) seeds treated with commercially available IAA at 50 µg/mL (positive control) to confirm that growth responses were consistent with IAA activity^36,37^. This design allows distinction between effects caused by IAA versus other metabolites potentially present in fungal culture filtrates. For each treatment and control, 30 seeds were used, arranged as three independent biological replicates of 10 seeds each. Seeds were placed on moistened sterile blotter papers in Petri dishes and incubated at 25 ± 2 °C for 5 days. Germination percentage, root length, shoot length, fresh and dry biomass, and seed vigor index (SVI) were determined following the method of Abdul-Baki and Anderson^38^. All experiments were performed in triplicate to ensure reproducibility, and data from independent biological replicates were analyzed to account for variability and strengthen the statistical validity of the results.

### Statistical analysis

Data were analyzed using SAS software. Results are expressed as mean ± standard deviation of triplicate measurements. Treatment means were compared using Duncan’s multiple range test at a 95% confidence level (*p* ≤ 0.05)^39^.

## Results

### Isolation and screening of IAA-producing fungi

Twenty fungal isolates were recovered from the rhizosphere soils of faba bean, wheat, garlic, and onion cultivated in Egypt. The isolates were purified, coded, and maintained for further analysis. Screening for indole-3-acetic acid (IAA) production revealed a wide variation among isolates (Table 1). Isolate FSA12 exhibited the highest IAA yield, producing 23.98 ± 1.98 µg/mL with a productivity rate of 2.99 µg/mL/day. The presence of IAA was confirmed using HPLC, where the sample chromatogram showed a retention time of 3.43 min, closely matching the IAA standard at 3.41 min (Figure S1), validating the identity of the detected compound.

**Table 1:** Screening of various isolates of fungi species for IAA production.

Isolate code	IAA concentration (µg/ml)	Productivity (µg/ml/day)
FSA_1_	11.22 ± 1.58 d	1.41
FSA_2_	14.51 ± 2.04 e	1.81
FSA_3_	9.87 ± 1.76 c	1.23
FSA_4_	12.38 ± 2.54 d	1.55
FSA_5_	1.25 ± 0.08 a	0.16
FSA_6_	-	-
FSA_7_	2.54 ± 0.84 a	0.32
FSA_8_	8.47 ± 1.78 c	1.06
FSA_9_	7.04 ± 0.55 b	0.88
FSA_10_	5.68 ± 1.05 b	0.71
FSA_11_	10.75 ± 1.89 d	1.34
FSA_12_	23.98 ± 1.98 g	2.99
FSA_13_	3.54 ± 0.99 a	0.44
FSA_14_	-	-
FSA_15_	13.22 ± 1.08 e	1.65
FSA_16_	-	-
FSA_17_	17.57 ± 1.44 f	2.19
FSA_18_	6.77 ± 1.08 b	0.85
FSA_19_	-	-
FSA_20_	4.78 ± 0.89 a	0.59

The results are presented as the means ± standard error. Each value is the mean of three replicates.

### Mycotoxin detection

Mycotoxin production was assessed using thin-layer chromatography (TLC) for the culture filtrate (Figure S2). The analysis, conducted at the Animal Health Research Institute (ARC), revealed the absence of detectable mycotoxins. This indicates that the selected isolate can be safely applied for agricultural purposes without posing toxicity risks to seeds or seedlings.

### Identification of the most efficient IAA-producing isolate

#### Morphological characterization

**Fig. 1:**
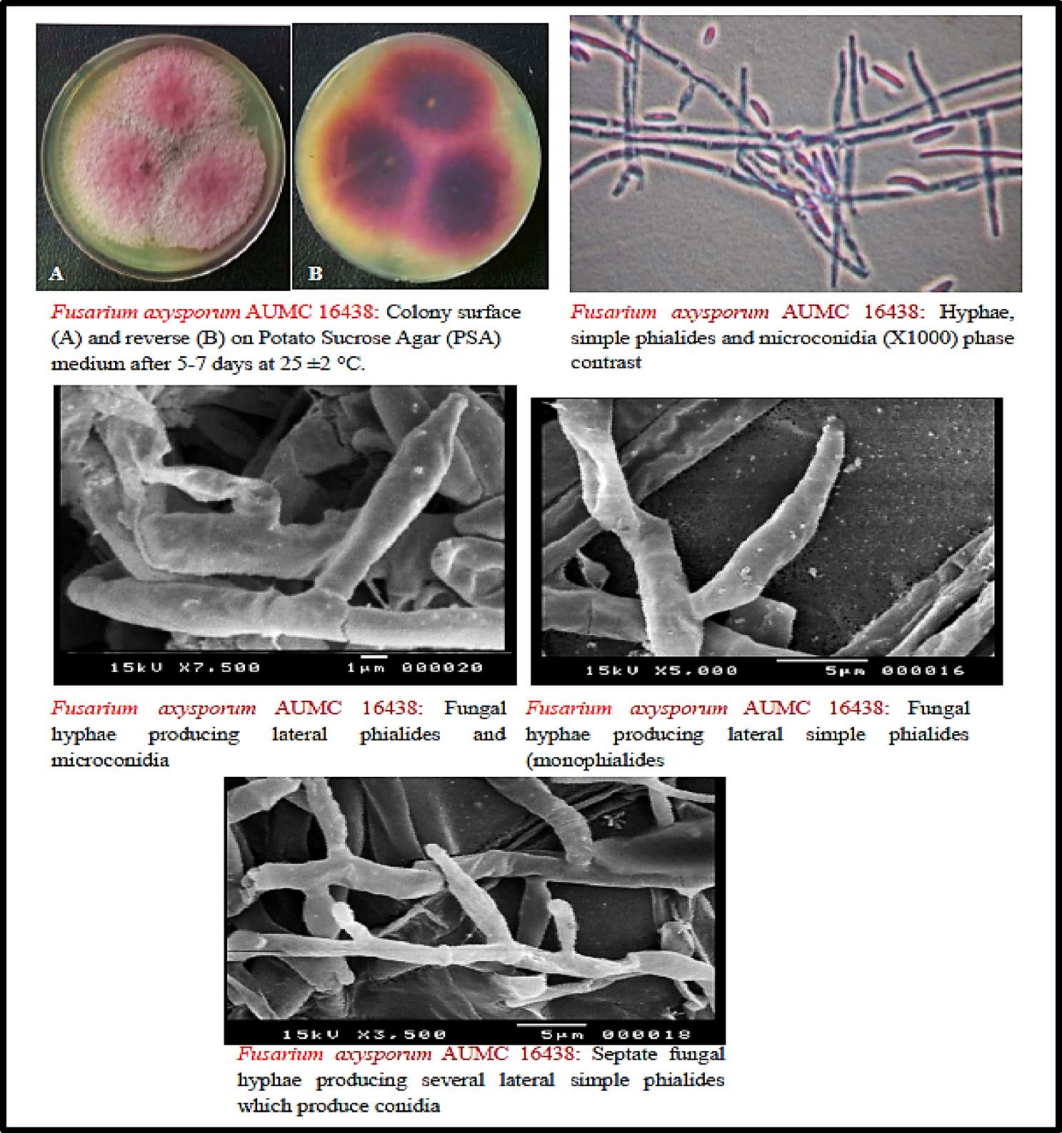
Morphological features of the fungal strain FSA12 (*F. oxysporum* AUMC 16438) based on visual and microscopic (light microscope and scanning electron microscopic) examination.

The most potent isolate, FSA12, was subjected to morphological and microscopic examination. After 5–7 days of incubation on PSA medium at 25 ± 2 °C, colonies displayed a felted, floccose texture with a reverse purple coloration and surface coloration ranging from white to pale apricot (Fig. 1).

Microscopically, conidiophores were hyaline, simple, and short, bearing conidial masses at the apex. Two types of conidia were observed:

- **Microconidia**: ellipsoidal, non-septate, formed in false heads on short monophialides.
- **Macroconidia**: sickle-shaped with slightly tapering apical cells and hooked basal cells.

Chlamydospores were globose, solitary or in pairs, and smooth- to rough-walled. Septate hyphae with lateral phialides producing chains of microconidia were consistently observed.

#### Molecular identification

**Fig. 2:**
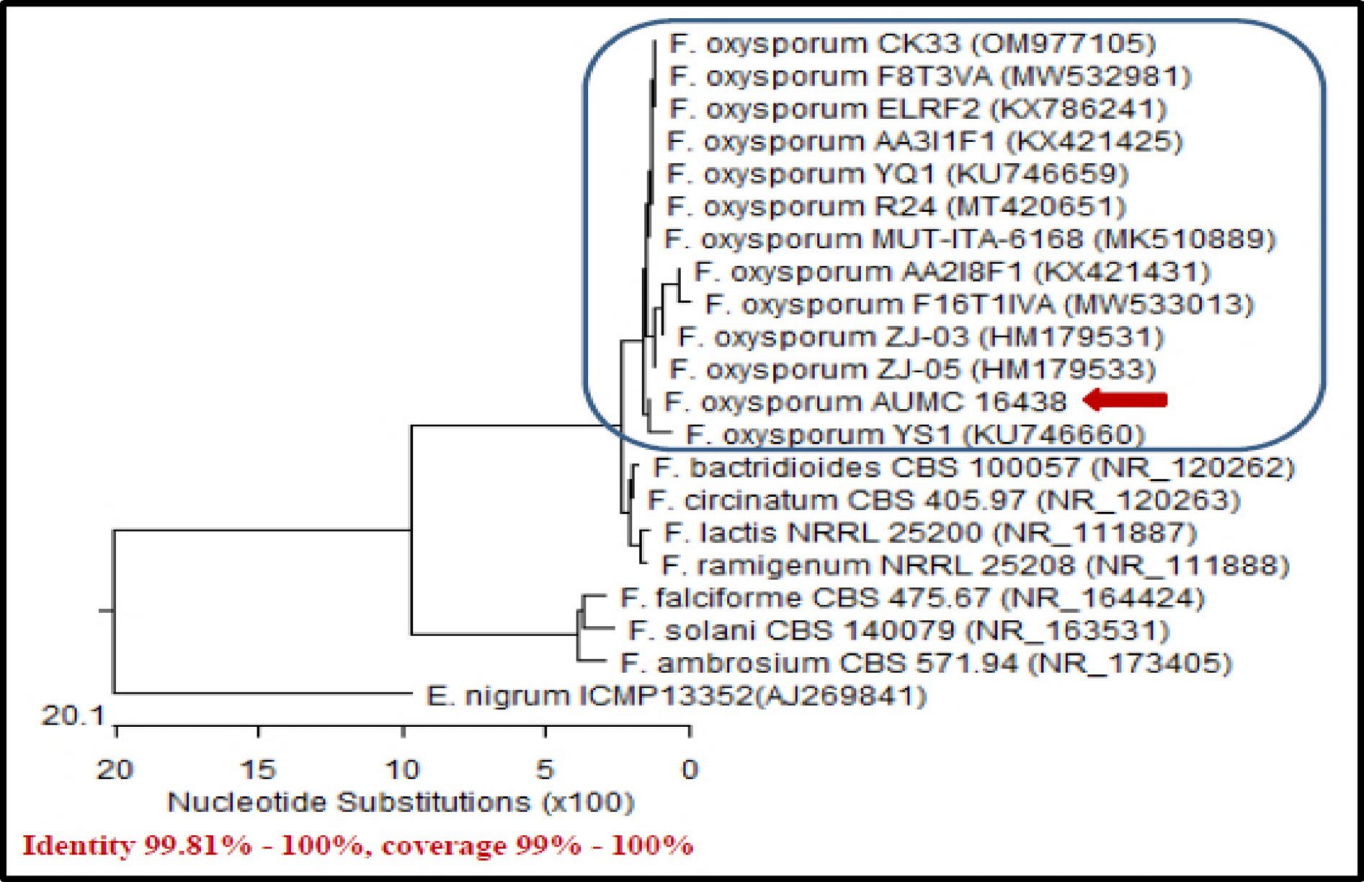
Phylogenetic tree based on ITS sequences showing relationships and position of the new isolate (*F. oxysporum* AUMC 16438 with accession number PP990199).

Amplification and sequencing of the ITS gene revealed that isolate FSA12 shares 99.81–100% similarity and 99–100% coverage with *Fusarium oxysporum*. The sequence was deposited in GenBank under accession number **PP990199**, confirming the isolate as *F. oxysporum* AUMC 16,438. A phylogenetic tree illustrating its taxonomic position is presented in Fig. 2.

### Optimization of culture conditions for IAA production

A series of one-factor-at-a-time (OFAT) experiments was conducted to optimize L-tryptophan concentration, incubation temperature, initial pH, incubation period, inoculum size, and the use of agro-industrial wastes as nutrient sources.

#### Effect of L-tryptophan concentration

**Fig. 3:**
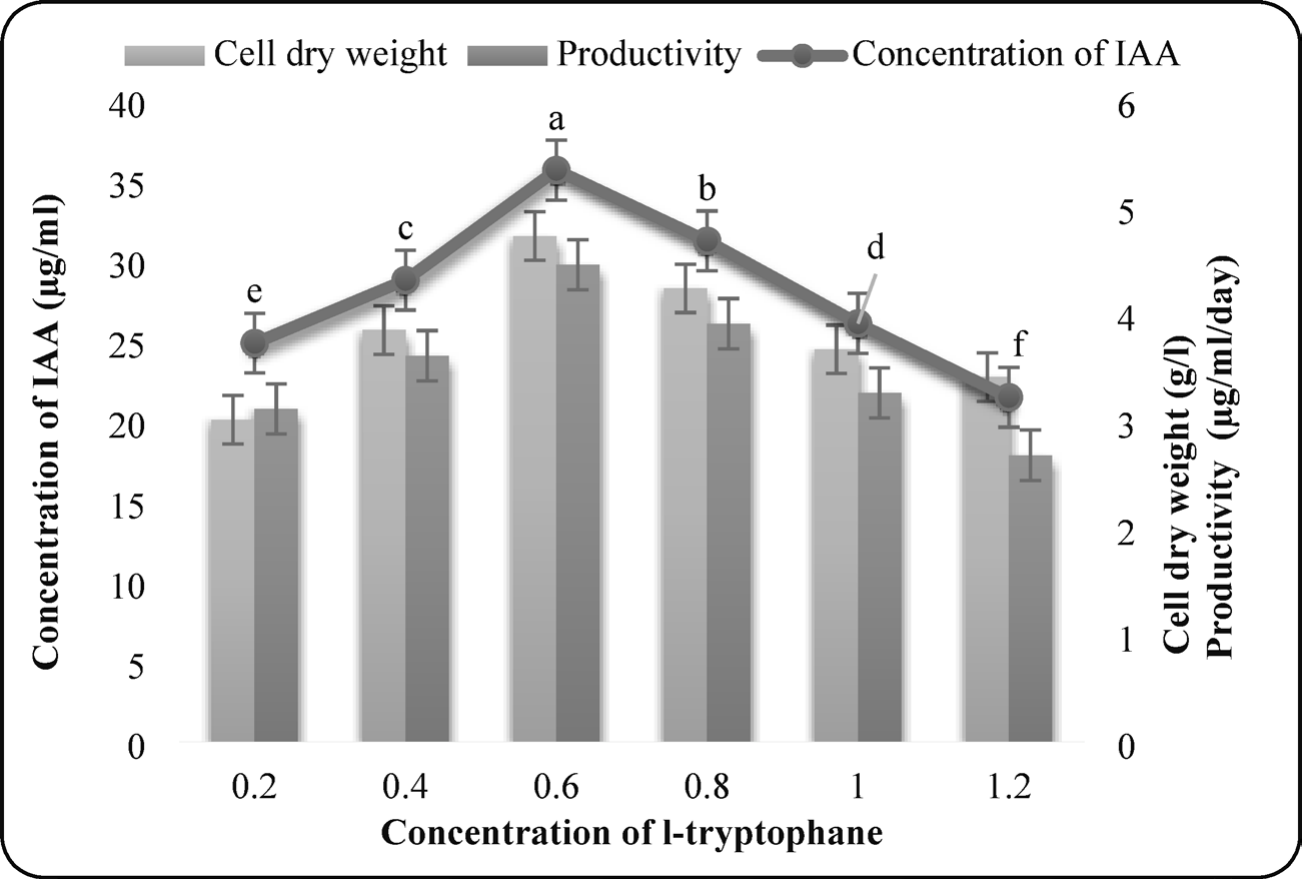
Effect of L-tryptophan concentration on IAA production by *F. oxysporum* AUMC 16,438. Each value is the mean of three replicates. Means designated by the same letter are not significant at the 5% level for each treatment.

L-tryptophan concentrations ranging from 0.2% to 1.2% were evaluated. Maximum IAA production (35.65 µg/mL), biomass yield (4.73 g/L), and productivity (4.46 µg/mL/day) were obtained at 0.6% L-tryptophan (Fig. 3). Higher concentrations negatively affected metabolite synthesis.

#### Effect of incubation temperature

**Fig. 4:**
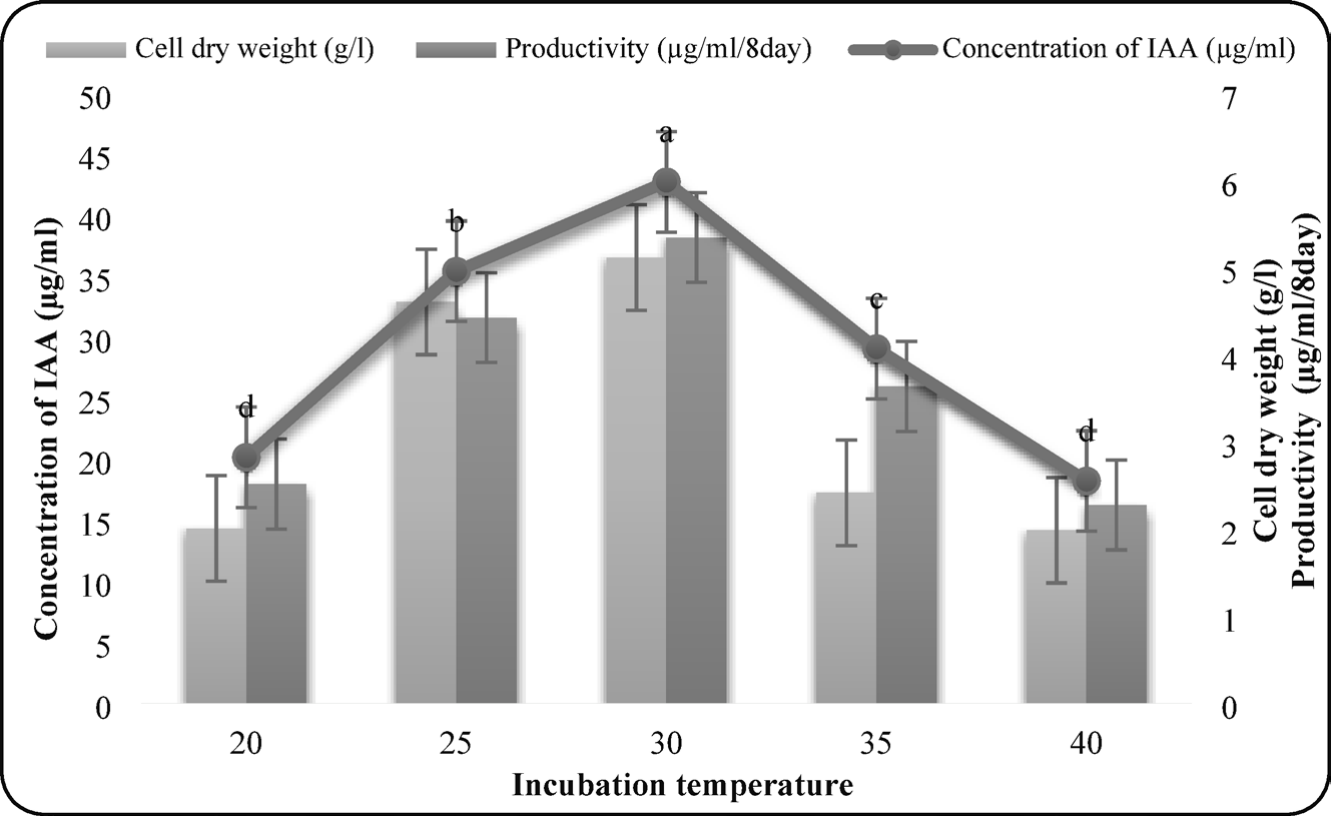
Effect of incubation temperature on IAA production by *F. oxysporum* AUMC 16,438. Each value is the mean of three replicates. Means designated by the same letter are not significant at the 5% level for each treatment.

Incubation temperatures of 20–40 °C were tested. Optimal IAA production (42.78 µg/mL), biomass (5.12 g/L), and productivity (5.35 µg/mL/day) were achieved at 30 °C (Fig. 4). Temperatures above or below this optimum significantly reduced IAA synthesis.

#### Effect of initial pH

**Fig. 5:**
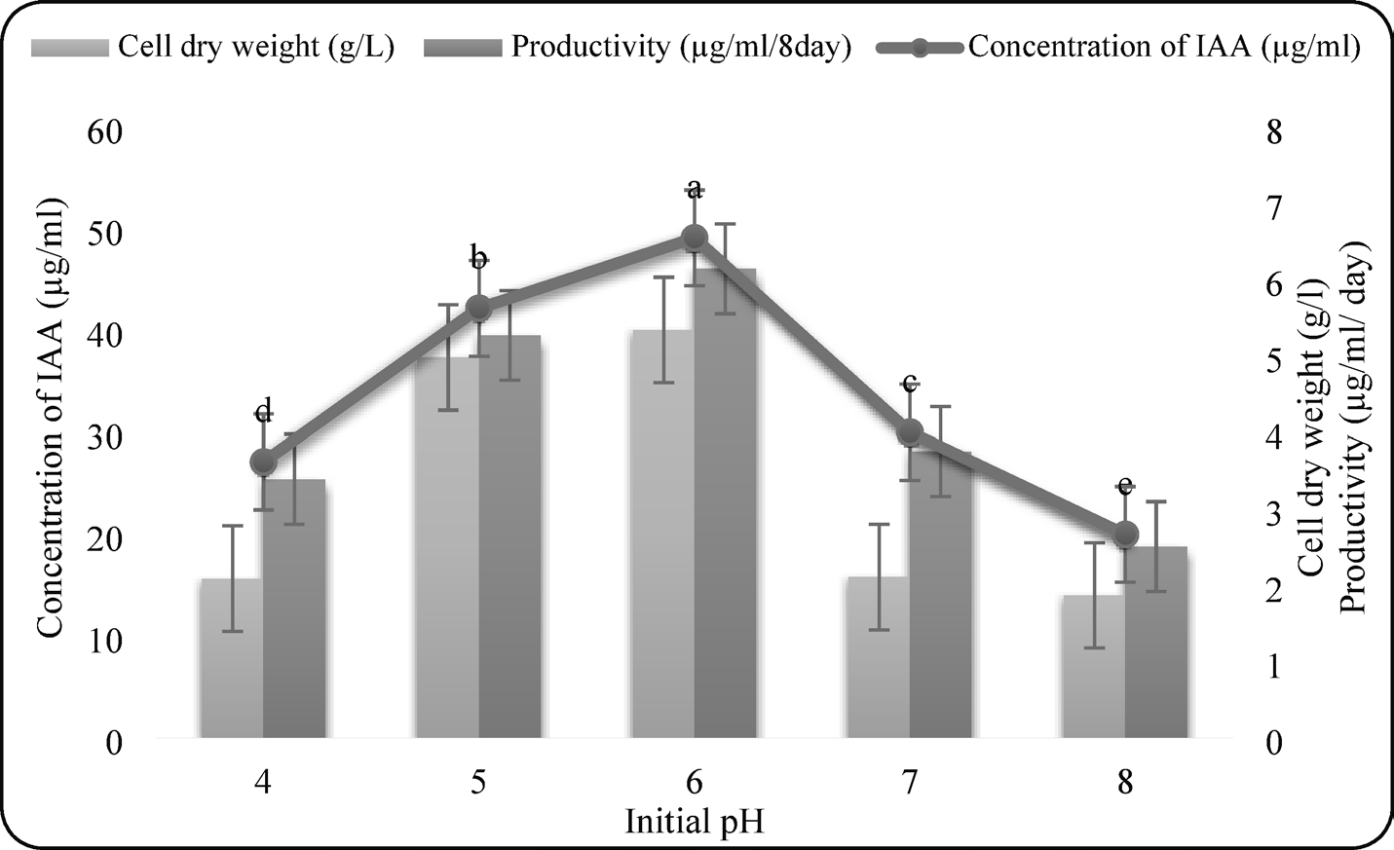
Effect of initial pH on IAA production by *F. oxysporum* AUMC 16,438. Each value is the mean of three replicates. Means designated by the same letter are not significant at the 5% level for each treatment.

The initial medium pH was adjusted between 4 and 8. Maximum IAA yield (49.09 µg/mL), cell dry weight (5.34 g/L), and productivity (6.14 µg/mL/day) occurred at pH 6 (Fig. 5), indicating the fungus favors slightly acidic conditions for auxin biosynthesis.

### Effect of incubation period

**Fig. 6:**
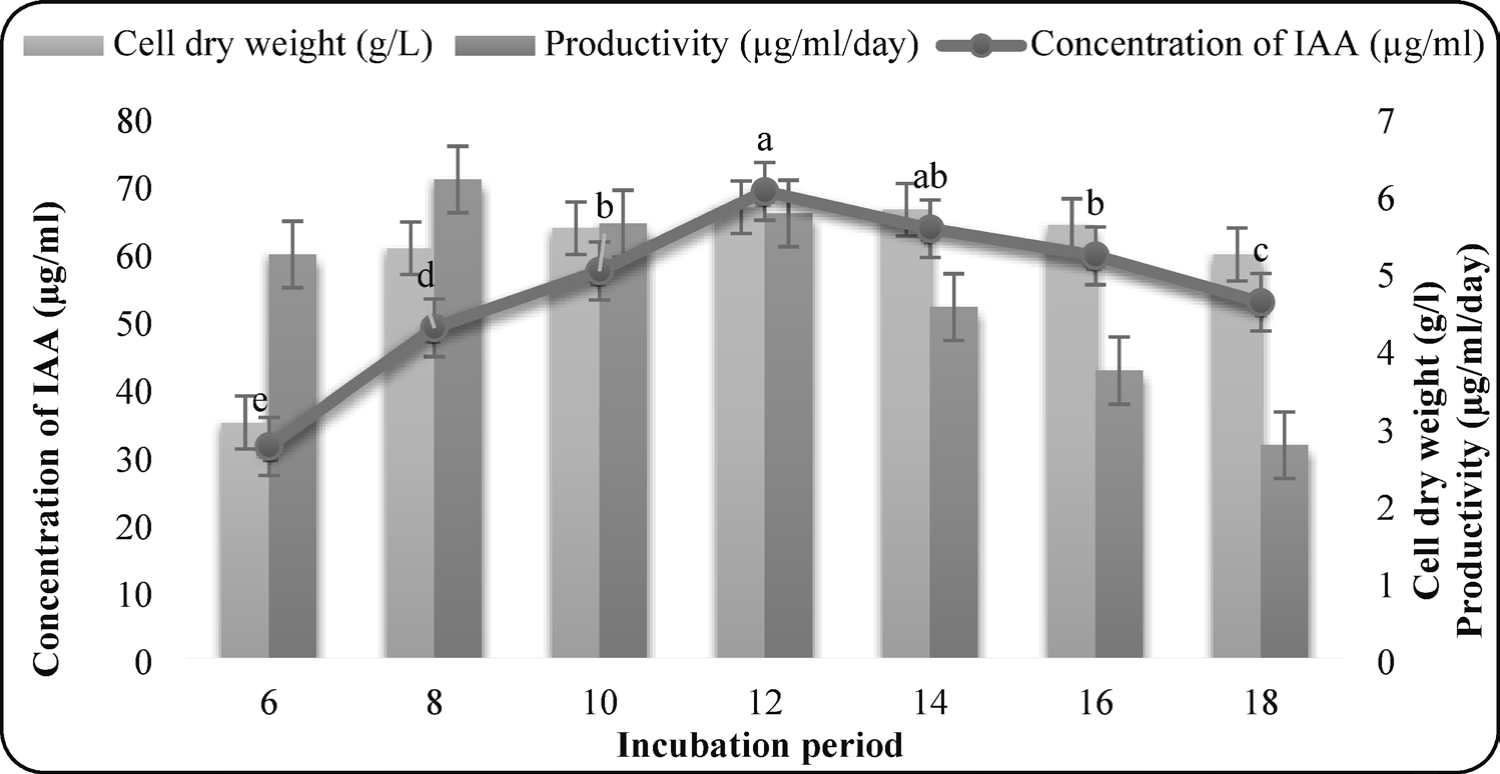
Effect of incubation period on IAA production by *F. oxysporum* AUMC 16,438. Each value is the mean of three replicates. Means designated by the same letter are not significant at the 5% level for each treatment.

IAA production increased progressively up to day 12, reaching its maximum (68.87 µg/mL) along with the highest biomass (5.82 g/L). In contrast, productivity peaked earlier on day 8 (6.18 µg/mL/day), suggesting that prolonged incubation favors accumulation rather than synthesis rate (Fig. 6).

### Effect of inoculum size

**Fig. 7:**
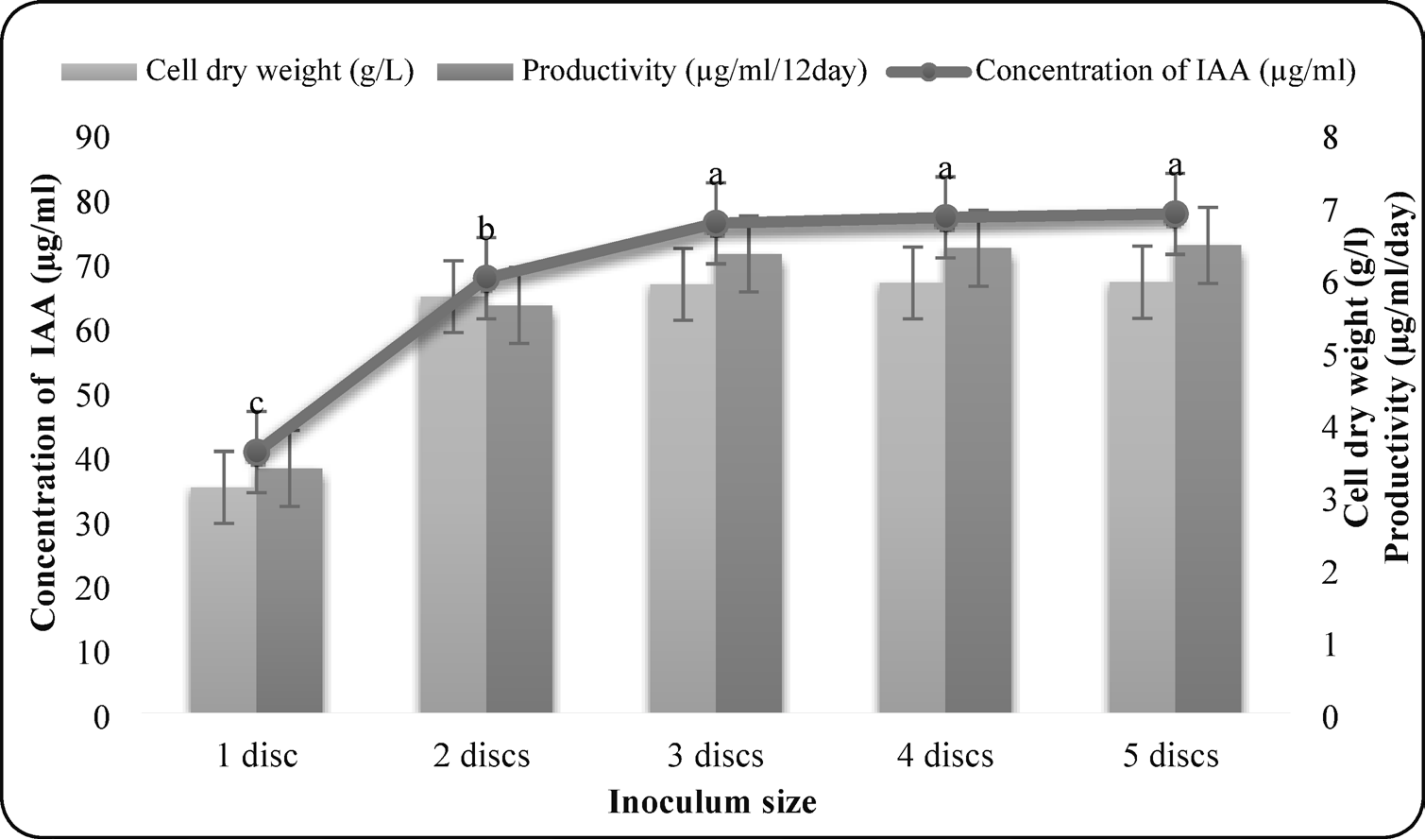
Effect of Inoculum size on IAA production by *F. oxysporum* AUMC 16,438. Each value is the mean of three replicates. Means designated by the same letter are not significant at the 5% level for each treatment.

Inoculum sizes of 1–5 fungal discs were tested. Three discs yielded the highest IAA concentration (75.97 µg/mL), biomass (5.91 g/L), and productivity (6.33 µg/mL/day). Larger inoculum sizes did not significantly enhance production (Fig. 7).

### Effect of agro-industrial wastes

**Fig. 8:**
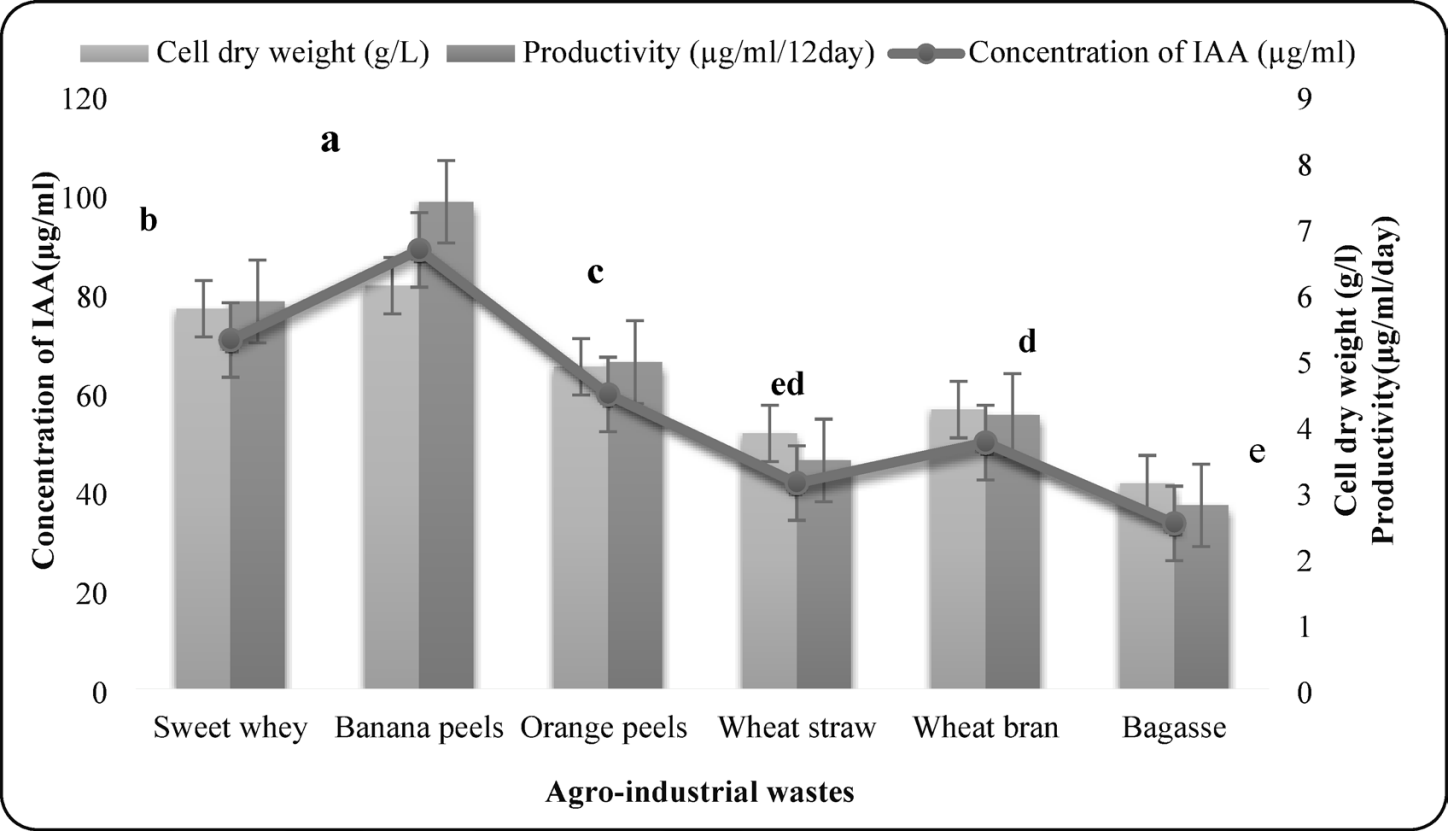
Effect of agro-industrial wastes on IAA production by *F. oxysporum* AUMC 16,438. Each value is the mean of three replicates. Means designated by the same letter are not significant at the 5% level for each treatment.

Six agro-industrial wastes (sweet whey, banana peels, orange peels, wheat straw, wheat bran, and bagasse) were examined as cost-effective carbon sources. All supported IAA production, but banana peels resulted in the highest IAA yield (88.67 µg/mL), biomass (6.11 g/L), and productivity (7.38 µg/mL/day) (Fig. 8). This enhancement is likely due to their natural L-tryptophan content.

### Application of produced IAA on wheat seed germination

The bioefficacy of the fungal-derived indole-3-acetic acid (IAA) was evaluated using wheat seeds as a model system. Seeds treated with the produced IAA exhibited significantly improved germination and early seedling growth compared with the untreated control (Table 2). Complete germination (100%) was achieved in the IAA-treated seeds, whereas only 70% germination was recorded in the control. In addition, the IAA application resulted in pronounced increases in root and shoot lengths, reaching 15 mm and 45 mm, respectively. Treated seedlings also showed higher fresh and dry biomass accumulation. Consequently, the vigor index of IAA-treated seeds (6000) was substantially higher than that of the control (1750), indicating a strong enhancement of early plant growth associated with the application of *F. oxysporum*–derived IAA.

**Table 2:** Effect of IAA produced by *F. oxysporum* AUMC 16,438 on wheat growth:.

Treatment	Germination (%)	Length of root (mm)	Length of shoot (mm)	Weight of fresh (g)	Weight of dry (g)	The vigor index
IAA production of *F. oxysporum*	100 a	15 ± 0.78 a	45 ± 0.69 a	2.2 ± 0.75 a	0.9 ± 0.82 a	6000 a
Control	70 b	5 ± 0.41 b	20 ± 0.67 b	1.3 ± 0.99 b	0.2 ± 0.94 b	1750 b

Each value is the mean of three replicates; Different letters indicate significant differences at *p* < 0.05, Means ± standard deviation (SD) are included for root, shoot, fresh weight, and dry weight.

## Discussion

Indole-3-acetic acid (IAA) is the predominant auxin regulating cell division, elongation, and differentiation, and it plays a central role in root architecture and overall plant development^5,40^. Microorganisms inhabiting the rhizosphere are recognized as important contributors to endogenous IAA pools, thereby indirectly modulating plant growth and stress adaptation^4,41^. In the present study, systematic screening of twenty fungal isolates recovered from rhizospheric soils in Qalyubia Governorate, Egypt, revealed considerable variability in IAA biosynthetic capacity, reflecting strain-specific metabolic potential. Among these isolates, FSA12 consistently exhibited superior IAA production, which was confirmed by HPLC analysis, indicating both quantitative reliability and biochemical specificity of the detected auxin^42^. Molecular identification based on ITS sequencing, together with morphological characterization, identified isolate FSA12 as *Fusarium oxysporum* AUMC 16,438 (GenBank accession no. PP990199). This finding is consistent with previous reports describing *F. oxysporum* as one of the most efficient fungal producers of IAA among rhizospheric fungi^43,44^. The IAA levels produced by strain AUMC 16,438 fall within, and in some cases exceed, ranges previously reported for *F. oxysporum* (100–140 mg/L)^45^, supporting the notion that this strain possesses a highly active tryptophan-dependent auxin biosynthesis pathway. Given that secondary metabolite production in *Fusarium* species may coincide with mycotoxin synthesis, assessing biosafety is critical for agricultural applications. Although thin-layer chromatography (TLC) did not detect mycotoxins under the tested conditions, it is acknowledged that TLC provides limited sensitivity. Therefore, while the absence of detectable toxins suggests a favorable safety profile for IAA production, future studies employing advanced analytical tools such as LC–MS/MS or ELISA are necessary to definitively confirm biosafety^46,47^. Nonetheless, the current findings indicate that under optimized fermentation conditions, *F. oxysporum* AUMC 16,438 can produce IAA without detectable mycotoxin accumulation. Optimization experiments revealed that nutritional and environmental parameters exerted strong regulatory effects on IAA biosynthesis, consistent with the dynamic nature of fungal secondary metabolism^48^. L-tryptophan supplementation significantly enhanced IAA production, confirming its role as a key precursor in tryptophan-dependent biosynthetic pathways^49^. The observed decline in IAA yield at higher tryptophan concentrations likely reflects feedback inhibition or metabolic imbalance, a phenomenon previously reported in both fungal and bacterial systems^50,51^. Similarly, the optimal temperature (30 °C) and initial pH (6.0) observed in this study are indicative of favorable enzyme activity and metabolic stability, conditions known to maximize auxin biosynthetic efficiency in filamentous fungi^7,12,52,53^. The temporal profile of IAA production showed a peak at 12–14 days, followed by a decline, which may be attributed to nutrient depletion, accumulation of inhibitory metabolites, or activation of IAA-degrading enzymes such as IAA oxidases^54,55^. Inoculum size also played a decisive role, with moderate biomass favoring metabolite accumulation, whereas excessive inoculation likely intensified competition for nutrients and oxygen, ultimately limiting IAA synthesis^56^. These findings underscore the importance of balanced growth conditions for maximizing metabolite yield. The use of agro-industrial wastes as alternative substrates further enhanced IAA production, with banana peels yielding the highest auxin concentration. This effect can be attributed to their intrinsic nutrient richness, particularly their content of tryptophan and soluble carbohydrates that support fungal growth and secondary metabolism^57^. Such results reinforce the feasibility of low-cost, sustainable bioprocessing strategies for biofertilizer development^58–60^. Importantly, the biologically produced IAA significantly improved wheat seed germination, seedling vigor, and biomass accumulation, demonstrating functional bioactivity beyond chemical quantification. These growth-promoting effects are consistent with IAA-mediated stimulation of root elongation, lateral root formation, and nutrient uptake efficiency^61,62^. Collectively, these results provide strong mechanistic and experimental evidence supporting *F. oxysporum* AUMC 16,438 as a promising and sustainable fungal candidate for IAA-based biofertilizer applications in agriculture.

## Conclusion

In this study, *F. oxysporum* AUMC 16,438 was identified as an efficient producer of indole-3-acetic acid (IAA). Optimization of nutritional and environmental parameters increased IAA production by approximately 3.7-fold, yielding a maximum concentration of 88.67 µg/mL. The culture filtrate was confirmed to be free of detectable mycotoxins, supporting the biosafety of the strain for agricultural use. Among the evaluated agro-industrial residues, banana peel was the most effective substrate for supporting IAA biosynthesis, offering a low-cost alternative to conventional carbon sources. The fungal-derived IAA significantly enhanced wheat seed germination and early seedling vigor. A key novelty of this work is the characterization of a non-pathogenic *F. oxysporum* strain capable of producing high levels of IAA while utilizing inexpensive agro-industrial waste materials. The integration of production optimization, biosafety verification, and functional bioassay evaluation provides a comprehensive assessment not commonly addressed in previous studies. These findings highlight the potential of *F. oxysporum* AUMC 16,438 as a sustainable plant growth–promoting agent and support its further development for agricultural applications.

## Supplementary Information

Below is the link to the electronic supplementary material.

Supplementary Material 1

## Data Availability

All data and materials are represented in the manuscript except the sequence of the molecular identification of the organism which can be accessed through the link [https://www.ncbi.nlm.nih.gov/nuccore/PP990199.1/].
