# Supplementary material for: Molecular identification and optimization of indole acetic acid production by Fusarium oxysporum AUMC 16,438 for biofertilizer application
Source: Sci Rep. 2026 Jan 27;16:3474. doi: 10.1038/s41598-026-35223-z (PMC12847781; doi:10.1038/s41598-026-35223-z)
Supplement: Supplementary file 1 — Supplementary Material 1 [file 41598_2026_35223_MOESM1_ESM.docx]

**
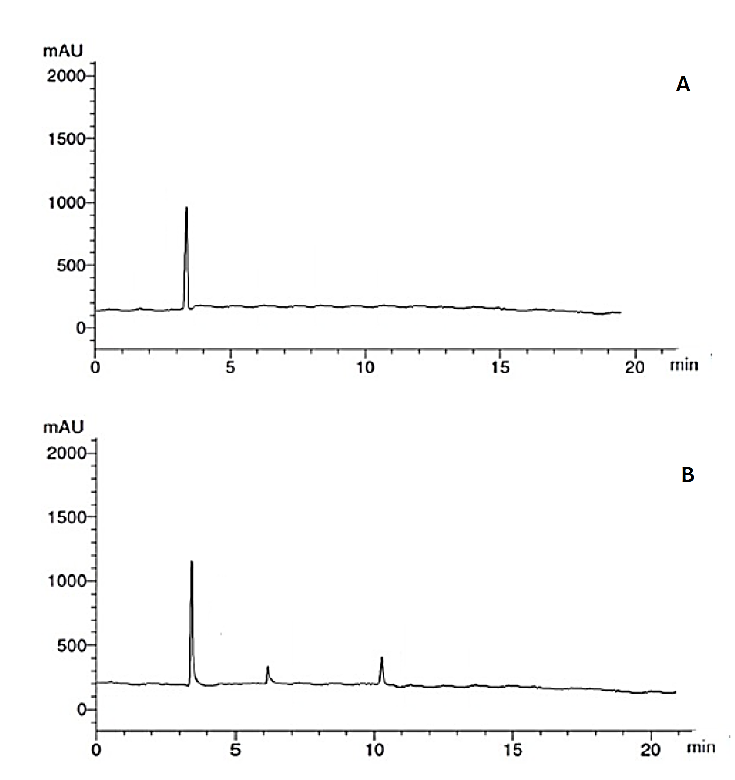
**

**Fig. S1** HPLC analysis of IAA produced by **A)** isolate FSA_12_ and **B**) standard IAA


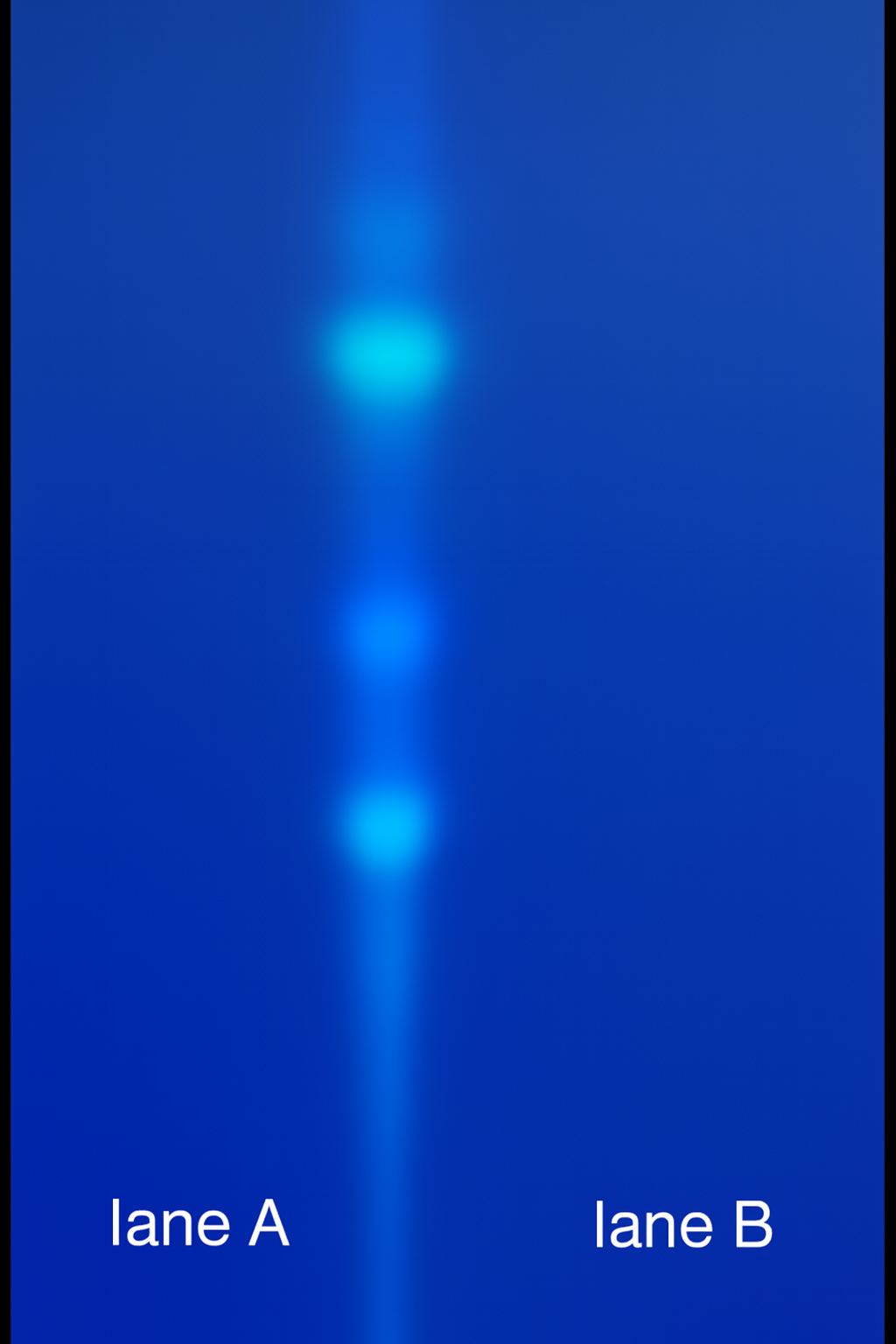


**Fig. S2** Thin layer chromatography (TLC); **lane A**: mycotoxin standard, **lane B:** Supernatant after growth of FSA_12_ isolate.
